# Risk calculation circuit abnormalities plus psychosocial risk variables predict problematic substance use in youth with externalizing disorders

**DOI:** 10.1038/s41386-026-02367-5

**Published:** 2026-02-26

**Authors:** Paola P. Mattey-Mora, Olivia K. Murray, Joseph Aloi, Mario Dzemidzic, Jaroslaw Harezlak, Leslie A. Hulvershorn

**Affiliations:** 1 Department of Psychiatry, Indiana University School of Medicine, Indianapolis, IN, USA.; 2 Adolescent Behavioral Health Research Program, Indiana University School of Medicine, Indianapolis, IN, USA.; 3 Medical Scientist Training Program, Indiana University School of Medicine, Indianapolis, IN, USA.; 4 Department of Neurology, Indiana University School of Medicine, Indianapolis, IN, USA.; 5 Department of Epidemiology and Biostatistics, Indiana University School of Public Health-Bloomington, Bloomington, IN, USA.

## Abstract

Prior research has identified brain regions associated with problematic substance use in youth, yet it remains unclear how neural processes during decision-making contribute to later drug use. Moreover, few studies have integrated psychosocial and environmental risk factors into predictive frameworks. This study investigated whether brain activation during risky decision-making in drug-naïve, high-risk children predicts problematic substance use during adolescence. Youth (*n* = 95; 64 male, mean baseline age=11.7 years) with externalizing disorders completed the Balloon Analogue Risk Task (BART) during functional MRI. Activation contrasts from six regions of interest, identified using a regularization-based feature selection method, were incorporated into cost-sensitive logistic regression models along with psychosocial and environmental variables, including family history of substance use, parental monitoring, and violence exposure. Models were adjusted for age at conversion to drug use, sex assigned at birth, and maternal education. Psychosocial-only factors showed fair predictive accuracy (AUC = 0.76; accuracy= 0.74) with good specificity and fair sensitivity. Neural activation–only models showed poor predictive accuracy (AUCs = 0.60–0.67; accuracy = 55–78%) with good specificity but limited sensitivity. Incorporating both psychosocial and neural factors substantially improved model performance (AUCs = 0.83–0.86; accuracy up to 82%), with fair sensitivity and good specificity in the adjusted models. These findings suggest that neural activity in regions involving risk evaluation, reward response, and sensory integration, together with relevant psychosocial factors predicts later problematic substance use, emphasizing the value of multidimensional models for early identification youth at elevated risk.

## INTRODUCTION

Behavioral treatments and prevention strategies for substance use disorders (SUDs) have largely been developed without incorporating addiction-relevant neural information [1], potentially limiting their effectiveness [2, 3]. Impaired ability to accurately calculate risk may be a driver for addictive behaviors [4–6]. Previously, we identified cross-sectional differences in brain activation within risk-calculation circuits among youth at increased risk for SUDs (e.g., family history, presence of disruptive behavior disorders [7, 8]). The current study extends these findings by examining whether risk-related neural activation predicts the initiation of problematic substance use (PSU) in a high-risk, substance-naïve sample of youth.

Adolescence is a critical period of neural, cognitive, and behavioral change associated with increased risk-taking [9]. Compared with children and adults, healthy adolescents show heightened activation in motivational and reward regions (e.g., nucleus accumbens, cortico-striatal circuits) and reduced activation in cognitive control regions (e.g., dorsolateral and inferior prefrontal cortex) [10], increasing vulnerability to risky behaviors such as substance use. Although early substance use elevates SUD risk, only some youth develop problematic use [11]. Accordingly, we focus on predicting problematic, rather than experimental, substance use from childhood measures.

Neural mechanisms underlying risky decision-making in adolescents with externalizing disorders (i.e., attention-deficit/hyperactivity disorder (ADHD), oppositional defiant disorder (ODD), and conduct disorder (CD)), a group at particularly increased SUD risk, remain underexplored. Some studies report greater activation in risk and reward processing (e.g. ventromedial prefrontal cortex, ventral striatum, anterior cingulate, and insula) regions during risky decision-making [8, 12] in youth with externalizing disorders [13–15]. Abnormalities in reward (e.g., nucleus accumbens, amygdala) and cognitive control regions (e.g., inferior frontal, anterior insula), during choice and reward anticipation, may contribute to PSU [16] in this population. However, the pathways through which neural differences in decision-making contribute to substance use outcomes remain unclear.

Psychosocial influences are known to impact the risk of onset of PSU [17, 18]. Low parental monitoring, parental SUDs, and family dysfunction all impact youth substance use [19–24]. Exposure to violence and peer influence further elevate substance use risk by promoting emotional dysregulation, increasing stress and anxiety, and altering brain circuits related to stress, reward processing and impulse control [23–28]. Drug availability may also be greater in locations with greater violence exposure and unstable family environments [6]. These factors may further influence neural development through stress-related and epigenetic mechanisms [7, 29–33], underscoring the need to integrate contextual risk alongside neurobiological measures.

Although brain activation during decision-making has been associated with risky substance use [4, 34, 35], most studies use cross-sectional designs and standard statistical approaches (descriptive and inferential). Predictive, machine learning-based modeling offers a robust and adaptable approach, capable of capturing complex relationships across biological, environmental, and demographic variables [36–38]. Longitudinal studies, including the Adolescent Brain Cognitive Development (ABCD) Study, have applied predictive approaches to associate neural predictors with later substance use in typically developing adolescents [39–41], but often lack direct measures of risk calculation. To address this gap, we aimed to determine whether neural activation during a risky decision-making task in drug-naïve children with externalizing disorders predicts later problematic substance use, while considering psychosocial factors typically absent from neuroimaging studies. We hypothesized that 1) neural activation explicit to risk calculation will prospectively predict problematic use; specifically, greater activation in regions of the reward (ventral striatum) and salience networks (anterior insula) and reduced activation in the central executive (dorsolateral prefrontal cortex) and default mode network (posterior cingulate cortex) during risky choices would be associated with greater risk of problematic substance use, and 2) multifactorial models combining neural, psychosocial factors would outperform the neural-only and psychosocial-only models. This approach provides an initial step toward identifying potential mechanisms underlying deficits in decision-making under risk that may ultimately inform targeted prevention and intervention strategies for vulnerable youth.

## METHODS AND MATERIALS

### Data source and study population

This observational study is part of an ongoing longitudinal project examining neural mechanisms of risky decision-making [42]. Participants (n = 192) were enrolled at age 11–12 and assessed every six months through 54 months. The final sample included 134 youth: 95 high-risk externalizing participants (67% male; mean age:15.04) and 39 healthy comparison youth (59.52% male; mean age: 14.09), the latter used for ROI identification.

High-risk participants included youth diagnosed with externalizing disorders according to DSM-5 criteria, specifically ADHD plus either ODD, CD or disruptive behavior disorder, unspecified. Exclusion criteria at enrollment included lack of English proficiency in children/caregivers; left-handedness [43]; prenatal substance exposure; current mood disorder (based on the DSM-5 criteria); lifetime history of psychotic, bipolar, or autism spectrum disorders; any prior substance use; neurological or debilitating medical conditions; IQ < 75; or magnetic resonance imaging (MRI) scanning contraindications [44, 45]. Written parental consent and youth assent were obtained prior to participation.

Inclusion required complete, high-quality neuroimaging data; the initial sample was reduced due to head motion, which is more common in individuals with ADHD [46, 47] (Fig. S1). Additional sample and methodological details have been previously published [8, 44, 45]. All procedures were conducted according to and approved by the Indiana University Institutional Review Board.

### Measures

#### Balloon analog risk task.

**Fig. 1:**
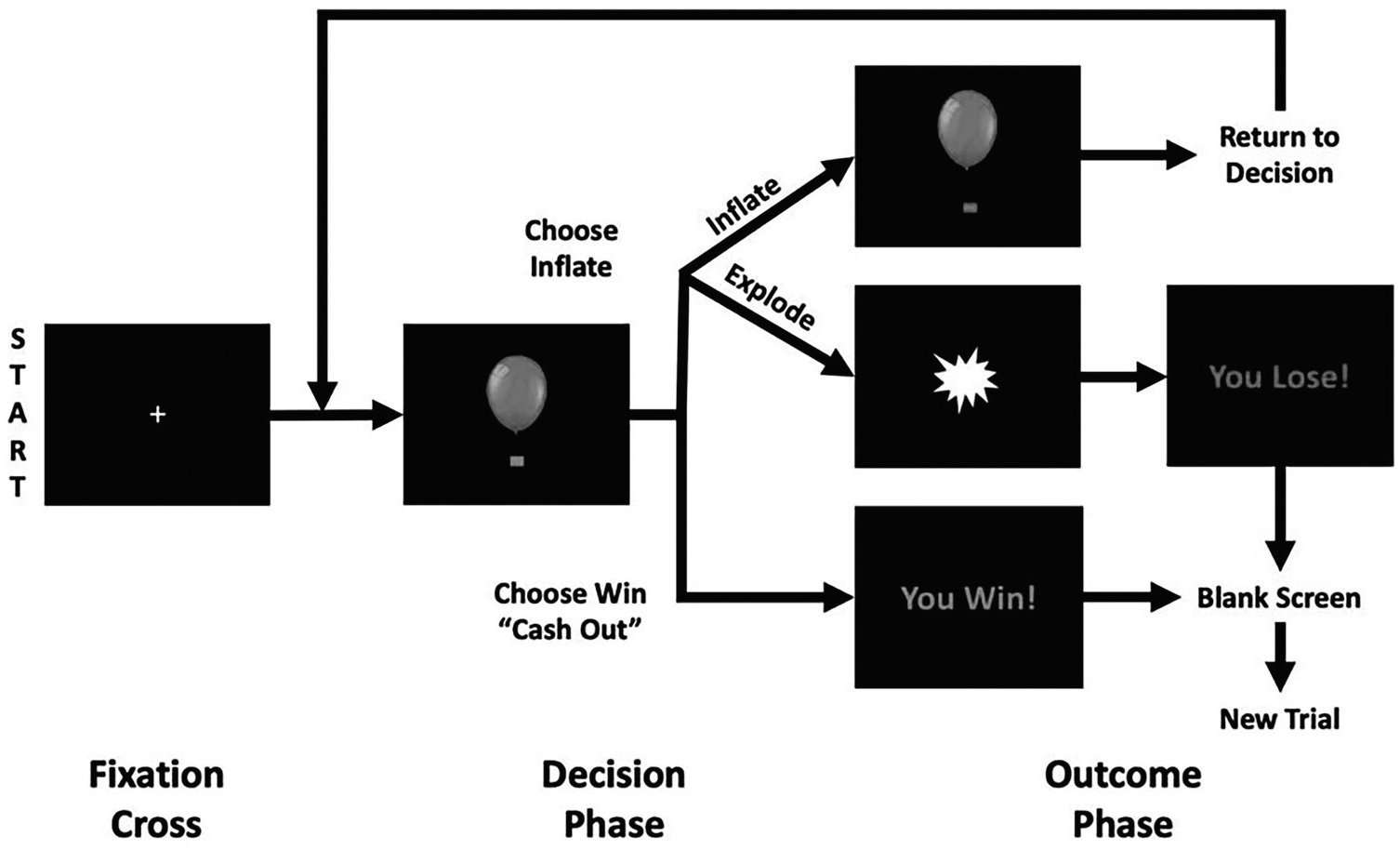
Balloon Analogue Risk Task diagram. Adapted from Aloi J, Kwon E, Hummer TA, *et al*. (2023). Frontiers Neuroimaging [7].

A well-established MRI-compatible version of the BART [48–50] was administered at baseline during MRI scanning to measure risky decision-making brain activation (Fig. 1). Additional descriptions of the BART task utilized here have been previously published [7, 8] and are available in the supplementary methods.

#### MRI data acquisition.

MRI scans were collected on a 3 T Siemens Prisma with a 32-channel head coil. Structural images were acquired with a high-resolution T1-weighted MPRAGE sequence (160 sagittal slices; voxel size = 1.05 × 1.05 × 1.2 mm^3^). Functional images used a multiband T2*-weighted EPI sequence (TR/TE = 1200/29 ms; flip angle = 65°; FOV = 220 × 220 mm^2^; matrix = 88 × 88; MB factor = 3; 54 axial slices; voxel size = 2.5 × 2.5 × 2.5 mm^3^; 400 volumes/run). Distortion correction employed two 16-sec phase-reversed spin-echo EPI scans (TR/TE = 1560/48 ms).

#### MRI preprocessing.

EPI distortions were corrected using spin-echo unwarping (FSL 6.0.1), followed by motion correction, 6-mm Gaussian smoothing, scaling (×100), and ICA-AROMA denoising (afni_proc.py). Images were normalized to T1 space and then to MNI space.

#### MRI processing.

First-level GLMs were run in AFNI, modeling six motion parameters, drift, and five task events (Choose Inflate, Choose Win, Outcome Inflate, Outcome Explode, Outcome Win), convolved with a double-gamma HRF. Contrasts (i.e., Choose Inflate–Win, Outcome Inflate-Win, Outcome Explode-Win, Outcome Explode-Inflate) with and without parametric modulation by explosion probability yielded beta maps. To avoid circularity [51], second-level GLMs identified significant clusters in an independent healthy control (HC) sample (*n* = 39) using 3dMVM, mapped to Schaefer 200-parcel and MNI atlases [52]. ROI activation values for EXT were extracted for predictive modeling. Multiple comparisons were corrected with 3dClustSim (10,000 iterations; voxel-wise *p* = 0.001; cluster size ≥30 voxels). Further details can be found in the supplementary material.

### Problematic substance use

Youth and parents completed the Substance Use Domain of the Drug Use Screening Inventory (revised; DUSI-R; [53]) with supplemented additional categories of newer substances. Follow-up assessments were conducted every six months after the baseline. Although follow-up occurred repeatedly, each youth contributed a single outcome indicating whether they transitioned into PSU at any point during the follow-up period or did not transition (all participants were drug naïve at baseline). The DUSI-R covered 25 substance classes/routes of administration, assessing monthly use frequency (0, 1–2, 3–9, 10–20, 21 +) and endorsement of substance-specific problems (e.g., “Have you missed out on activities because you spent too much time or money on this substance?”). Additionally, youth also provided samples for a 5-panel urine screen and an alcohol breathalyzer (Uritox, LLC, Toledo, OH). Urine screens were coded dichotomously, with conservative coding applied when discrepancies arose (i.e., positive urine results or higher self-reported use took precedence). PSU was defined as meeting ≥1 of the following: frequent use (≥10 times/month for a substance), ≥2 DSM-5–based consequences, unsafe use (e.g., legal problems, injury), or use of a potentially lethal substance (e.g., inhalants, cocaine, opioids; excluding alcohol, tobacco, cannabis, vaping). Participants not meeting criteria were classified as drug naïve.

### Psychosocial factors

Psychosocial factors included family history of substance use disorders (FH +) (yes/no), parental monitoring, and exposure to violence. Parental monitoring was assessed using an adaptation of the Silverberg Parental Monitoring Scale [54]), focusing on how often parents knew their child’s whereabouts after school (1 = never, 5 = always). Exposure to violence was assessed with two subscales of the Screen for Violence Exposure (SAVE) [55]: Indirect Exposure (e.g., witnessing violence or hearing about violent events), and Traumatic Exposure (e.g., being a direct victim of violent acts). Higher scores indicate greater violence exposure. Traumatic violence exposure was greater in youth who developed PSU (Table 1).

### Covariates

Covariates included age of onset of problematic substance use event (or censored at last report for youth without PSU), given increased SUD risk with earlier ages [11], sex assigned at birth, and maternal education as a proxy for the child’s socio-economic status (i.e., mother’s education: high-school, 2-year degree, 4-year degree, some or completed graduate or professional school).

### Statistical analysis

Brain activation and environmental factors were compared between participants who became problematic substance users during the follow up period and those who remained substance use naïve or used in less risky ways. T-tests, Wilcoxon’s rank sum test, chi-squared test, and Fisher’s exact test were utilized according to the data type and distribution. Statistical significance was set at α < 0.05. All statistical analyses were done in R version 4.1.0 [56].

In this study, we used a data-driven, predictive modeling approach designed to identify patterns in neural and psychosocial features that best distinguished youth who later developed PSU from those who did not. Elastic net regularization was first applied for variable selection on MRI features that were identified from the healthy control group, resulting in 89 selected features. A cost-sensitive logistic regression model was then fitted to predict problematic substance use (a binary outcome). The analysis was developed in several stages; 1) a psychosocial-only model, which included only family history of substance use disorders, parental monitoring, and exposure to violence; 2) a psychosocial model adjusted for the confounders: age of onset, sex assigned at birth, and mother’s education (to quantify the predictive contribution of psychosocial factors in isolation); 3) individual brain-only models, which included only the identified individual neural activation contrast parcels from the risky decision-making (BART) task; 4) individual models with brain predictors plus the addition of psychosocial factors; and finally, these same models adjusted for the potential confounders.

To address class imbalance in the data (i.e., 26 youth who developed PSU (PSU +) vs 69 youth without PSU (PSU-)), we applied class weighting by assigning higher penalties for misclassifying the minority class (i.e., users). Sample weights were computed based on the inverse prevalence of each class and incorporated into the logistic regression. The model was trained using a seeded 10-fold cross-validation framework to ensure robustness. For model comparison, we also computed a null model that included no predictors and assigned each participant the base-rate probability of problematic substance use; performance metrics were calculated using the same cross-validation framework.

Model performance was optimized for the area under the receiver operating characteristic curve (AUC). Class probabilities were extracted, and thresholds for classifying individuals as PSU+ youth were selected using Youden’s Index (range = 0.46–0.61). The thresholds were applied to classify participants. Additionally, we evaluated secondary performance metrics, including accuracy (overall correct classification), sensitivity (correctly identifying true PSU+ youth), and specificity (correctly identifying true PSU- youth). Model improvement in discrimination performance was assessed using the DeLong’s test. The R packages “pROC” [57] and “caret” [58] were used.

Data missingness for parental monitoring, violence exposure, and maternal education was handled with multiple imputations utilizing predictive mean matching with the other covariates (MICE package in R; [59]). A more detailed statistical analysis description can be found in the supplementary material.

## RESULTS

Predictive analyses were restricted to high-risk youth with externalizing disorders whose imaging data passed quality control (*n* = 95) [60]. Participants excluded for excessive motion (*n* = 50) included PSU+ youth (*n* = 3), substance using PSU− youth (n = 16), and substance-naïve PSU− youth (*n* = 31), resulting in the absence of non-using youth in the final analytic sample. Healthy comparison youth were used only for region-of-interest definition.

At follow-up, 27.4% of participants (*n* = 26) met criteria for problematic substance use at a mean age of 15.5 years. Family history of SUD was reported by 49.5% of the sample and was more prevalent among PSU+ youth than PSU− youth (73.1% vs. 40.6%, *p* = 0.01). PSU+ youth also showed lower parental monitoring (*p* < 0.01) and greater exposure to traumatic and indirect violence (*p* = 0.03; *p* = 0.02; Table 1).

**Fig. 2:**
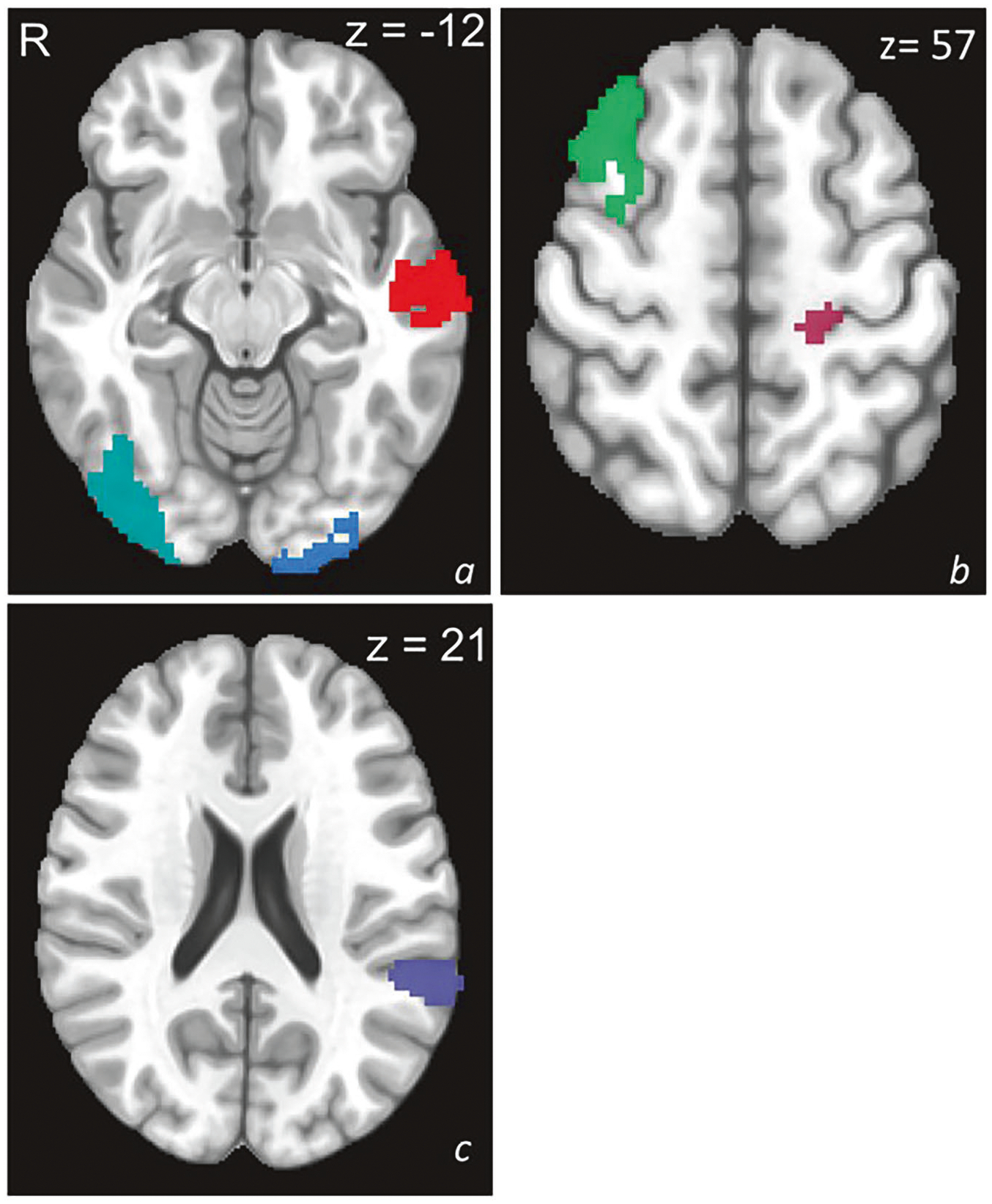
Significant predictive parcels. Identified significant predictive parcels: (**a**) left middle temporal gyrus (red), left middle occipital gyrus (blue), right inferior occipital gyrus (teal); (**b**) left postcentral gyrus (mauve), right middle frontal gyrus (green); (**c**) left temporal parietal junction (purple).

From BART task contrasts in substance-naïve healthy controls, 87 parcels were identified as potential ROIs based on relevance to risky decision-making (Table S2). Penalized feature reduction yielded six parcels spanning default mode, executive control, attention, visual, and somatomotor networks for predictive modeling across the Choose Inflate–Win and Outcome Explode–Win contrasts (Table 2; Fig. 2).

### Predictive model with psychosocial factors only

The model including family history of SUD, parental monitoring, and exposure to violence, showed fair predictive performance (AUC = 0.76; Accuracy = 0.74, CI = 0.64–0.82; Table 3). Nonetheless, its predictive performance did not differ from the null model (AUC = 0.50, Accuracy = 0.72, CI = 0.65–0.81).

### Predictive models with neural activation only

Five of six regions of interest showed associations with PSU. However, neural activation–only models demonstrated overall poor predictive performance. During the choice phase (Choose Inflate–Win modulated), models based on activation in the left middle temporal gyrus (AUC = 0.66; Accuracy = 0.74, CI = 0.64–0.82), temporoparietal junction (AUC = 0.62; Accuracy = 0.73, CI = 0.63–0.81), right middle frontal gyrus (AUC = 0.60; Accuracy = 0.55, CI = 0.44–0.65), and left postcentral gyrus (AUC = 0.67; Accuracy = 0.78, CI = 0.68–0.86) did not outperform the null model. Similarly, during the outcome phase (Outcome Explode–Win), activation in the left middle occipital (AUC = 0.62; Accuracy = 0.63, CI = 0.53–0.73) and right inferior occipital gyri (AUC = 0.60; Accuracy = 0.72, CI = 0.62–0.81) showed limited discrimination relative to the null model. All unadjusted models demonstrated fair-acceptable specificity (i.e., correctly identifying individuals who will not engage in problematic substance use), they exhibited poor-fair sensitivity (i.e., correctly identifying individuals who will engage in problematic substance use; Table 3).

### Predictive models with neural activation and psychosocial factors

After incorporating neural activation, family history of SUD, parental monitoring, and exposure to violence, all models showed statistically significant improvements over neural activation-only models in predicting problematic substance use (Table 3). Both accuracy and AUC also increased.

In the Choose Inflate-Choose Win modulated regressor contrasts, the adjusted models for the left middle temporal gyrus (Accuracy = 0.76, CI = 0.66–0.84, AUC = 0.81), left temporal parietal junction (Accuracy = 0.69, CI = 0.59–0.79, AUC = 0.78), and right middle frontal gyrus (Accuracy = 0.74, CI = 0.64–0.82, AUC = 0.78) demonstrated significant improvements over the models with only neural activation. Similarly, in the Outcome Explode-Outcome Win unmodulated contrasts, models for the left middle occipital gyrus (Accuracy = 0.64, CI = 0.54–0.74, AUC = 0.80) and right inferior occipital gyrus (Accuracy = 0.63, CI = 0.53–0.73, AUC = 0.79) showed better predictive capacity. However, for the left postcentral gyrus activation, psychosocial factors did not significantly improve predictive performance. Specificity remained acceptable across all models. Sensitivity was fair for all the models, when compared to the models with neural activation only (Table 3).

When models were further adjusted for age, sex, and maternal education, predictive performance remained consistent. For example, the adjusted model for the left middle temporal gyrus (Accuracy = 0.81, CI = 0.72–0.89, AUC = 0.86) and the left temporoparietal junction (Accuracy = 0.81, CI = 0.71–0.88, AUC = 0.83) continued to discriminate PSU+ youth from PSU- youth. Similarly, models for the right middle frontal gyrus (Accuracy = 0.80, CI = 0.71–0.86, AUC = 0.83), left middle occipital gyrus (Accuracy = 0.81, CI = 0.72–0.88, AUC = 0.85), and right inferior occipital gyrus (Accuracy = 0.82, CI = 0.73–0.89, AUC = 0.84) remained robust. This adjustment did not alter predictive performance, as models were not statistically different from the unadjusted combined models (Table 3).

## DISCUSSION

This study examined whether childhood brain activation during risky decision-making, combined with psychosocial factors, predicts PSU in high-risk adolescents. Across brain regions, adjusted models combining neural and psychosocial predictors showed good performance (accuracy: 80–82%; AUC: 83–86%), high specificity (81–86%) and fair sensitivity (73–81%). Performance was comparable to prior predictive neuroimaging studies in high-risk youth (accuracy: 62–85%, sensitivity and specificity: 66–75%) [61–63], but to our knowledge, this study is the first to apply predictive modeling to brain imaging during risky decision-making in high-risk youth.

As expected, neural activation alone provided limited prediction, as decision-making is only one domain relevant to SUD risk [64]. However, when incorporating psychosocial factors, the predictive capacity improved substantially, reinforcing the notion that risky substance use is best understood through a multifactorial lens. Neural predictors provide an insight into which decision-making and reward-related regions contribute to vulnerability to problematic substance use. As shown in the results, neural activation contributes primarily to improvements in the overall discriminative performance rather than altering the classification threshold that drives the secondary parameters. And, even when predictive gains are modest, incorporating neural activation improves interpretation of risk pathways and strengthens multimodal models of adolescent substance use. These neural patterns may also help identify potential targets for neuromodulation (e.g., TMS), supporting future intervention development.

Although psychosocial factors alone showed fair performance (AUC = 0.76; accuracy = 74%), prediction did not significantly outperform the null model, indicating that while these variables are important, they are insufficient without the addition of neural predictors. Consistent with prior work, integrating neurobiological and psychosocial risk (e.g., parental monitoring, drug availability, etc.) result in stronger prediction than either alone [62, 63]. Family history, violence exposure (also a potential proxy for drug availability and acceptability of drug use), and parental monitoring significantly improved prediction, highlighting their influence on neurodevelopment and substance use trajectories. The addition of these factors and the changes in the model performance metrics is consistent with prior evidence that drug use-relevant environmental factors shape neurodevelopment, decision-making, and substance use trajectories, with evidence that these factors account for substantial variance in adolescent substance-use risk; whereas neural activation tends to provide smaller incremental improvements in prediction [39, 65]. Further adjustment for demographic variables did not meaningfully improve performance, suggesting that the interplay between neural and environmental factors is the critical driver.

Although the combined model performed best, incremental effect-size gains from the addition of neural predictors were modest, consistent with prior work showing that psychosocial and demographic factors contribute more strongly to adolescent substance use risk, with neural activation providing smaller, incremental effects [39, 65]. Neural measures therefore tend to provide smaller contributions, enhancing mechanistic interpretation rather than yielding large gains in predictive performance.

During the choice phase (Choose Inflate–Win), modulated activation in the lateral temporal cortex (LTC), dorsolateral prefrontal cortex (dlPFC), and temporoparietal junction (TPJ) predicted problematic substance use, with primary somatosensory cortex (S1) activation showing marginal effects (Fig. 2). During the outcome phase (Outcome Explode–Win), visual cortex (VC) activation also predicted problematic substance use. These regions represent the medial temporal, central executive, and sensory networks implicated in externalizing behavior, decision-making, and addiction.

These findings are consistent with existing literature suggesting that alterations in the middle temporal gyrus, part of the LTC, a key region within the default mode network, may be associated with immediate gratification and diminished activation during inhibitory control, thereby increasing susceptibility to substance use [66, 67]. The dlPFC (which includes the middle frontal gyrus and a hub of the central executive network) has been associated with processing negative consequences, emotional regulation, and perceptual decision-making which play a role in risk evaluation [68, 69]. Decreased activation in the TPJ, part of the dorsal attention network, has been associated with deficits in decision-making and impulsivity, potentially contributing to poor risk evaluation despite awareness of negative consequences, further increasing the risk of substance use [14, 70].

Furthermore, decreased activation in the left postcentral gyrus (part of S1) during the choice phase was identified as a predictor for PSU. Although traditionally linked to motor and somatosensory functions, the postcentral gyrus contributes to decision-making by integrating sensory feedback and evaluating potential outcomes under risk or uncertainty, facilitating adaptive decision-making processes [71, 72].

Activation in the inferior and middle occipital gyri, components of the central visual network, were also predictors of PSU. Although the occipital cortex has been associated with visual processing, recent evidence has suggested that the visual central network plays a key role in decision-making, particularly visual cues, perception, and attention selection [73]. The inferior occipital gyrus, a key structure within the ventral visual pathway, has been implicated in visually guided decision-making and processing risk-related visual stimuli [74]. In the current study, increased activation in this region may have been recruited in response to salient negative outcomes, such as the balloon explosion [75, 76]. Similarly, the middle portion of the occipital gyrus has been associated with compensatory mechanisms to maintain stability during gain vs loss processing, as well as attention modulation and risk perception through visual cues [77].

Several limitations should be noted. First, generalizability is limited because we focused only on high-risk youth with externalizing disorders. While this design was appropriate for identifying early predictors among those most vulnerable to SUDs, exclusion of youth with subthreshold symptoms reduced variance and may have introduced bias [78, 79]. In addition, non-random motion-related exclusions, which disproportionately affected non–substance-using participants, together with ~30% attrition during follow-up, further constrained representativeness, introduced selection bias, and limit generalizability. Although standard motion thresholds and class-weighted models were applied to mitigate these effects, some selection bias may remain, underscoring the need for replication in larger samples. Second, we used regularization to reduce variance and false positives, reliance on a data-driven method may have failed to capture other relevant predictors, as the feature-selection process may identify associations specific to this sample. Third, predictive accuracy was likely limited by the modest sample size and number of participants who developed PSU. While elastic net regularization, class weighting, and 10-fold cross-validation mitigated these concerns, some instability in model estimates may remain. Fourth, feature selection and modeling were performed within the same dataset, raising overfitting concerns, although regularization reduced this concern by coefficients shrinkage, reducing model complexity. Fifth, assessments of problematic use during the COVID-19 pandemic relied primarily on self- and parent-report, which can be biased, although urine screening was obtained when possible. Finally, because neural activation was measured in late childhood, while follow-up occurred in mid-adolescence (16–17 years), some neural–behavioral associations may not yet have fully emerged, potentially attenuating observed effects, becoming clearer later in life.

Despite these limitations, the study design preserved temporality, capturing the initiation of problematic substance use in a fully substance-naïve sample. Circularity was minimized by defining regions of interest in an independent, same-age healthy cohort. The application of cost-sensitive machine learning to integrate neural and psychosocial factors is a novel approach that improves robustness, prevents overfitting, inaccurate effect size estimates, and lack of reproducibility, compared to prior methods. Finally, the focus on youth with externalizing disorders targets a population at elevated risk for SUDs and of high relevance for prevention efforts.

Our models highlight how neural activation during risky decision-making, coupled with relevant psychosocial factors can identify youth with problematic substance use. Importantly, activation in cortical regions supporting reward sensitivity (lTC), inhibitory control (TPJ), risk assessment and self-control (dlPFC), sensory feedback (S1), and visual processing, rather than traditional circuits alone (e.g. reward), were predictive of problematic use only when combined with contextual risks such as family history, parental monitoring, and violence exposure. Neither neural nor psychosocial factors alone were sufficient, underscoring the interactive nature of vulnerability.

These findings advance mechanistic understanding of SUD risk processes, though the practical use of fMRI for large-scale prediction remains limited due to cost, accessibility, and the predictive power of psychosocial factors alone. Thus, the current contribution lies in identifying novel mechanisms underlying propensity for SUDs such as the importance of risk calculation and the sensory aspects of perceiving and processing risk, particularly in the context of youth experiencing high levels of adversity. Although the combined models demonstrated meaningful discrimination, findings should be interpreted cautiously given the data-driven design, modest sample size, and that results are specific to our sample. These results provide a foundation for replication and refinement in larger, independent samples. Future longitudinal studies with multidimensional designs, tracking neural change over time and validated through cross-validation or independent replication, are required to confirm the robustness of our results and guide interventions.

## Supplementary Material

Supplementary Material

**Supplementary information** The online version contains supplementary material available at https://doi.org/10.1038/s41386-026-02367-5.

## Figures and Tables

**Table 1.** Baseline characteristics of the participants with externalizing disorders^a^.

	Total Sample of participants with Externalizing Disorders (n = 95)	Substance Naive/youth without PSU (*n* = 69)	Youth who developed PSU (*n* = 26)	*p*-value
Age onset of PSU^b^	15.04 (1.18)	14.88 (1.14)	15.47 (1.20)	**0.03**
Sex (male) (%)	64 (67.00)	47 (68.10)	17 (65.40)	1.00
Family History SUD (yes) (%)	47 (49.50)	28 (40.60)	19 (73.10)	**0.01**
Parental Monitoring (% who always monitor their kids)	84 (88.40)	65 (95.70)	18 (69.20)	**<0.01**
Traumatic Violence (SAVE mean score)	14.08 (4.83)	13.43 (2.96)	15.81 (7.73)	**0.03**
Indirect Violence (SAVE mean score)	28.57 (11.12)	26.93 (10.69)	32.92 (11.25)	**0.02**
Maternal Education (%)				0.87
At Least High School/GED	12 (12.60)	4 (15.40)	8 (11.60)	
Some or Graduate College	54 (56.80)	14 (53.80)	40 (58.00)	
Some or Complete graduate Degree or Similar	29 (30.50)	8 (30.80)	21 (30.40)	

a Values mean (SD) or n (%) unless indicated otherwise. *SUD* substance use disorder. Significant value at *p* < 0.05 are highlighted in bold. *PSU* problematic substance use. *SAVE* Screen for Violence Exposure.

b Age for non-substance/naïve users was calculate at last follow-up.

**Table 2.** Selected MRI features at a 90% agreement from penalization.

Contrast	Preliminary identified Peak MNI Coordinate [x,y,z] (mm^3^)	Atlas	Yeo 17 Network	Parcel	Parcel voxel size
Choose Inflate – Choose Win modulated	−58, −10, −15	Schaefer200	Default Mode B	Left Middle Temporal Gyrus	336
	35, 14, 57		Control B	Right Middle Frontal Gyrus	326
	−20, −27, 64		Somatomotor A	Left Postcentral Gyrus	82
	−60, −41, 19		Dorsal Attention B	Left Temporal Parietal Junction	169
Outcome Explode – Outcome Win unmodulated	−24, −99, −10	Schaefer200	Visual Central	Left Middle Occipital Gyrus	250
	38, −83, −9	MNI-Colin27	Visual Central	Right Inferior Occipital Gyrus	491

**Table 3.** Prediction summaries for the classification models between brain activation, psychosocial factors,^a^ and problematic substance use.

	Model		AUC	Accuracy (95%CI)	Sensitivity	Specificity	Model improvement^c^ (p-value)
	Psychosocial Only^a^		0.76	0.74 (0.64–0.82)	0.65	0.77	---
	Psychosocial Adjusted^b^		0.81	0.79 (0.69–0.87)	0.73	0.81	0.22
Choose Inflate – Choose Win modulated	Left Middle Temporal Gyrus	Brain only	0.66	0.74 (0.64–0.82)	0.46	0.84	---
		Brain + Psychosocial	0.81	0.76 (0.66–0.84)	0.81	0.74	0.02
		Brain + Psychosocial adjusted^b^	0.86	0.81 (0.72–0.88)	0.73	0.84	0.15
	Right Middle Frontal Gyrus	Brain only	0.60	0.55 (0.44–0.65)	0.77	0.46	---
		Brain + Psychosocial	0.78	0.74 (0.64–0.82)	0.77	0.72	0.01
		Brain + Psychosocial adjusted^b^	0.83	0.80 (0.71–0.86)	0.77	0.81	0.27
	Left Postcentral Gyrus	Brain only	0.67	0.78 (0.68–0.86)	0.42	0.91	---
		Brain + Psychosocial	0.80	0.76 (0.66–0.84)	0.69	0.78	0.06
		Brain + Psychosocial adjusted^b^	0.83	0.82 (0.73–0.89)	0.73	0.86	0.41
	Left Temporal Parietal Junction	Brain only	0.62	0.73 (0.63–0.81)	0.35	0.87	---
		Brain + Psychosocial	0.78	0.69 (0.59–0.79)	0.85	0.64	0.01
		Brain + Psychosocial adjusted^b^	0.83	0.81 (0.71–0.88)	0.73	0.84	0.17
Outcome Explode – Outcome Win unmodulated	Left Middle Occipital Gyrus	Brain only	0.62	0.63 (0.53–0.73)	0.58	0.65	---
		Brain + Psychosocial	0.80	0.64 (0.54–0.74)	0.92	0.54	0.02
		Brain + Psychosocial adjusted^b^	0.85	0.81 (0.72–0.88)	0.81	0.82	0.17
	Right Inferior Occipital Gyrus	Brain only	0.60	0.72 (0.62–0.81)	0.42	0.84	---
		Brain + Psychosocial	0.79	0.63 (0.53–0.73)	0.92	0.52	0.01
		Brain + Psychosocial adjusted^b^	0.84	0.82 (0.73–0.89)	0.73	0.86	0.22

*AUC* Area Under the Receiver Operator Curve.

a Environmental and psychosocial factors included: family history of substance use, parental monitoring, and indirect and traumatic violence.

b Models adjusted for age of conversion, sex assigned at birth, and mother’s education.

c *P*-values corresponding to model improvement on performance with Delong test. Unadjusted multifactorial models are compared to brain only models. Multifactorial adjusted models are compared between unadjusted models. Statistically significant *p*- values are highlighted in bold.

## Data Availability

The data used in this study are drawn from an ongoing longitudinal study following youth in the United States and will be deposited in a national data repository in 2026.
