# Supplementary material for: Risk calculation circuit abnormalities plus psychosocial risk variables predict problematic substance use in youth with externalizing disorders
Source: Neuropsychopharmacology. 2026 Feb 26;51(7):1335–44. doi: 10.1038/s41386-026-02367-5 (PMC13047751; doi:10.1038/s41386-026-02367-5)
Supplement: Supplementary file 1 — Supplementary Material [file 41386_2026_2367_MOESM1_ESM.docx]

**Supplementary Material**

Methods

Measures

*Balloon Analog Task*

Participants were instructed to inflate virtual balloons for cash and were told that the larger the un-popped balloon, the larger the cash amount they would be paid. Balloons that popped had no cash value, thus youth needed to balance the number and size of the balloons that they chose to inflate or “cash out.” Youth were told that they would be paid in cash immediately after the scan, with the goal being to earn as much money as possible. Each balloon had a maximum inflation limit of 12 inflations and participants were administered three 8-minute runs of the task. Before in-scanner BART administration, participants practiced the task on a computer outside of the scanner to ensure they understood the task.

Task related events were modeled as the time point at which a participant pressed the button to either inflate the balloon (Choose Inflate) or stop inflating and win the money (Choose Win). Outcome events were aligned to the time point when the balloon was successfully inflated (Outcome Inflate) or exploded (Outcome Explode).

*MRI Data Acquisition*

Scans were conducted using a 3T Siemens Prisma MRI scanner with a 32-channel head coil. Co-registration and normalization of functional image volumes to Montreal Neurological Institute space were completed using a high-resolution 3D magnetization prepared rapid gradient echo (MPRAGE; 160 sagittal slices; 1.05×1.05×1.2 mm^3^ voxels) structural scan. Blood Oxygenation Level Dependent (BOLD) functional MRI was performed using a T2*-weighted multiband (MB) echo-planar imaging (EPI) sequence (Gradient Echo; Repetition/Echo Time (TR/TE) 1200/29ms; flip angle 65^o^; field-of-view 220x220mm^2^; matrix 88x88, MB slice acceleration factor 3; 54 axial slices; voxel size 2.5×2.5×2.5mm^3^; 400 BOLD volumes in each functional scan). A pair of 16-second phase-reversed spin echo EPI scans (TR/TE 1560/48ms; 3 A-to-P and 3 P-to-A phase direction volumes) was acquired prior to functional imaging at the same locations and voxel size as the BOLD EPI acquisition.

*MRI Data Preprocessing*

Susceptibility-induced distortions and magnetic field inhomogeneities in all EPI scans were corrected through spin-echo unwarping, using the FMRIB Software library (FSL version 6.0.1). Using afni_proc.py functional images were motion-corrected to the initial volume of the first functional run and spatially smoothed with a 6mm full-width at half-maximum Gaussian kernel. The EPI data underwent time-series normalization to a T1-weighted image, and these voxel values were multiplied by 100 for each voxel values to achieve better comparisons across voxels and subjects. Finally, an unsupervised ICA-AROMA classifier was applied for noise and component identification in each individual’s data to ensure robust denoising for further analysis.

*MRI Data Processing*

First-level GLM analyses were conducted in AFNI [1]. The three runs were concatenated prior to first-level modeling. Six motion parameters, six motion derivatives, and a scanner drift term were modeled. Initial regressors were created by convolving the train of stimulus events with a double-gamma hemodynamic-response function with a first-order temporal derivative to create a model BOLD response time series for each condition. The regressors included five task-related events (Choose Inflate, Choose Win, Outcome Inflate, Outcome Explode, and Outcome Win). Sequentially, the contrasts Choose Inflate–Win, Outcome Explode–Inflate, Outcome Inflate-Win, and Outcome Explode-Win were calculated. First level analysis was conducted at the individual level.

The task-related regressors and contrasts were parametrically modulated by the balloon explosion probability for each pump. This procedure generated unmodulated b-coefficients/t-statistics for each voxel and regressor as well b-coefficients/t-statistics for each voxel and regressor modulated by explosion probabilities. Parametric modulation improves construct validity by modeling neural responses to continuous changes in objective risk, rather than relying solely on discrete event types. Prior work has shown that modulated activation during the BART better indexes risk evaluation and individual differences in decision-making than unmodulated task activation, as it captures neural responses to increasing risk in key valuation and control regions[2].

For the second-level GLMs on BOLD response, first contrast clusters (choose inflate–win, outcome explode–inflate, outcome inflate-win, and outcome explode-win) with significant activation/responses during the task in the overall sample relative to baseline, were identified from a distinct sample of 39 healthy control youth (age 11.38, n= 25 males; without psychiatric disorders or familial SUDs), using AFNI’s 3dMVM[3]. These clusters served to locate the activation peaks for the *a priori* regions of interest / parcels identified later in the Schaefer cortical (200 parcels) [4]and Montreal Neurological Institute (MNI) standard space [5] atlases. This approach minimized circularity, guaranteeing independence in the statistical inference, and allows a better interpretation of abnormal brain activation in the group with externalizing disorders, as the parcels were established from a normative pattern. To assess whether HC-derived ROIs were appropriate for cluster identification, we compared baseline peak activation values between HC and EXT, across all identified ROIs (n=87) for the choose inflate–win, outcome explode–inflate, outcome inflate-win, and outcome explode-win contrasts, using Welch two-sample t-tests and Hedges’ g (with FDR correction). No differences were detected, and effect sizes were uniformly small (median |d| = 0.14), indicating no meaningful baseline differences in ROI activation. We also compared variability using Levene’s test, which showed no significant variance differences, with similar variability across groups (median EXT/HC SD ratio = 1.05). These results support the use of HC-derived ROIs in the EXT sample without introducing bias.

Finally, average activation of these parcels was later calculated in the participants with externalizing behaviors, for the prediction modeling. Figure 3 shows the imaging pre-processing and processing steps.

Multiple comparison correction was performed using a spatial clustering operation in AFNI’s 3dClustSim utilizing the autocorrelation function (-acf) with 10,000 Monte Carlo simulations for the whole-brain analysis. Spatial autocorrelation was estimated using the residuals from the individual-level GLM analyses. The initial voxel-wise threshold was set at p=.001. The voxel threshold was set at 30 voxels minimum.

Problematic Substance use

Although a non-problematic use group was conceptually possible, all participants who reported any substance use met at least one criterion for problematic substance use; therefore, the sample did not include substance-exposed youth without PSU. Excluded and included participants did not differ on demographic or behavioral variables (p > 0.05), except for substance use (7% vs. 27%, respectively) (Table S1). The motion-related exclusions were not completely random and likely reflect an overlap in motion and behavioral differences associated with early substance initiation[6].

Statistical analysis

*Feature Selection*

Before the predictive model fitting, regularization was conducted to accomplish variable reduction among the MRI features, with 89 features identified *a priori.* This regularization enabled the selection of potential predictors from high-dimension data[7]. Additionally, regularization helps address multicollinearity, prevents overfitting, improves generalization, and enhances exposure selection[8]. First, ten-fold cross-validation was performed to select the regularization tuning parameter lambda (λ), which controls the overall strength of the penalty term (α), controlling for the shrinkage of the data values toward the central point. Lambda was selected by the minimum cross-validated error approach. Once the penalization terms were determined, elastic net regressions (regularization method) were conducted for variable reduction. This procedure was repeated 100 times to ensure the reproducibility of the regression outcomes, to generate stable results and to minimize spurious findings. The “*glmnet*” R package was used for this purpose[9].

*Classification*

Classification modeling for the development of problematic substance use based on 6 contrast parcels for neural activation during decision-making, as well as with relevant environmental influences ((age of risky substance use onset, family history of substance use, parental monitoring, and indirect and traumatic violence) was performed with a cost-sensitive logistic regression approach. The binary outcome variable (Problematic Use vs. Non-use) was derived from self-reported substance use and recoded into categorical factors for analysis. Given the class imbalance in our sample, we applied class weighting to penalize misclassification of the minority class (Users). Sample weights were calculated based on the inverse prevalence of each class and were incorporated into the logistic regression model. Model training was implemented using a seeded model with 10-fold cross-validation. Additionally, performance was optimized for the area under the receiver operating characteristic curve (AUC), and class probabilities were extracted to enable threshold tuning, which corresponded to the highest Youden’s Index. The thresholds (range: 0.46-0.61) were subsequently applied to classify participants, and model performance was evaluated using confusion matrix metrics, including accuracy (correct classification of a large proportion of cases; range: 0-100%), sensitivity (correctly identify individuals who truly will develop risky substance use; range: 0-100%), and specificity (correctly identify individuals who truly will not develop risky substance use; range: 0-100%). Additionally, to determine whether the inclusion of the psychosocial/environmental predictors significantly improved model performance, we compared the AUCs of the competing models using DeLong’s test for two correlated ROC curves[10]. This method provides a non-parametric approach to test whether the difference in AUCs is statistically significant when between models. All analyses were conducted using the packages “pROC” [11]and “caret”[12], with statistical significance set at *p* < 0.05.

Figure S1. Consort Diagram.

Table S1. Comparison of baseline characteristics between included and excluded participants with externalizing disorders based on neuroimaging data quality

|  | Included Participants  (N=95) | Excluded Participants  (N=50) | p-value |
| --- | --- | --- | --- |
| Age^2^ | 15.04 (1.18) | 15.01 (1.31) | 0.86 |
| Sex (Male) (%) | 63 (67.00) | 34 (67.00) | 0.79 |
| Family History SUD (Yes) (%) | 47 (49.50) | 27 (54.00) | 0.73 |
| Parental Monitoring (% who always monitor their children) | 84 (88.40) | 43 (86.00) | 0.88 |
| Traumatic Violence (SAVE mean score) | 14.08 (4.83) | 13.43 (2.96) | 0.76 |
| Indirect Violence (SAVE mean score) | 28.57 (11.12) | 26.93 (10.69) | 0.61 |
| Maternal Education (%) |  |  | 0.73 |
| At Least High School/GED | 12 (12.60) | 8 (16.00) |  |
| Some or Graduate College | 53 (55.80) | 29 (58.00) |  |
| Some/Complete Graduate Degree or Similar | 30 (31.60) | 13 (26.00) |  |
| Problematic Substance Use (Yes) (%) | 26 (27.40) | 3(6.00) | **<0.01** |

^1^Values mean (SD) or n (%) unless indicated otherwise. SUD= substance use disorder. Significant value at *p*<0.05 are highlighted in bold. PSU= problematic substance use. SAVE= Screen for Violence Exposure.

^2^Age for non-substance/naïve users was calculate at last follow-up.

Table S2. Significant ROI selection from the Second level GLMs on BOLD response of the healthy control individuals

|  | Preliminary Clusters | Peak MNI Coordinate (mm^3^) | | |
| --- | --- | --- | --- | --- |
|  |  | x | y | z |
| Choose Inflate - Choose Win modulated | Right Middle Temporal Gyrus | -55 | 67 | 3 |
|  | Left Middle Frontal Gyrus | 30 | -56 | 18 |
|  | Right Middle Frontal Gyrus | -50 | -38 | 18 |
|  | Right Superior Medial Gyrus | -13 | -38 | 41 |
|  | Right Inferior Frontal Gyrus (p. Orbitalis) | -30 | -41 | -17 |
|  | Right Superior Temporal Gyrus | -58 | 7 | 6 |
|  | Right Middle Frontal Gyrus | -30 | -8 | 56 |
|  | Right Mid Orbital Gyrus | -13 | -48 | -7 |
|  | Left Superior Temporal Gyrus | 65 | 47 | 18 |
|  | Left SMA | 5 | -18 | 53 |
|  | Left Putamen | 28 | 19 | 8 |
|  | Left Middle Temporal Gyrus | 60 | 24 | -12 |
|  | Left Inferior Frontal Gyrus (p. Triangularis) | 38 | -16 | 31 |
|  | Right Superior Temporal Gyrus | -68 | 2 | -7 |
|  | Left SMA | 3 | 12 | 61 |
|  | Right Middle Frontal Gyrus | -53 | -18 | 41 |
|  | Right Superior Temporal Gyrus | -48 | 42 | 18 |
|  | Right Middle Frontal Gyrus | -35 | 4 | 58 |
|  | Left Inferior Frontal Gyrus (p. Orbitalis) | 33 | -38 | -17 |
|  | Left Precentral Gyrus | 23 | 29 | 58 |
|  | Left Thalamus | 18 | 27 | 11 |
|  | Right Heschls Gyrus | -43 | 27 | 13 |
|  | Right Rolandic Operculum | -60 | -6 | 13 |
|  | Right Inferior Frontal Gyrus (p. Opercularis) | -53 | -6 | 23 |
|  | Right Cerebellum Lobule IX | -10 | 47 | -45 |
| Choose Inflate - Choose Win unmodulated | Right Inferior Parietal Lobule | -43 | 42 | 48 |
|  | Right Cuneus | -13 | 82 | 33 |
|  | Left Inferior Parietal Lobule | 30 | 52 | 48 |
|  | Right Lingual Gyrus | -18 | 67 | -5 |
|  | Left Lingual Gyrus | 8 | 79 | 1 |
|  | Right Middle Frontal Gyrus | -50 | -38 | 33 |
|  | Left Inferior Frontal Gyrus (p. Triangularis) | 48 | -31 | 23 |
|  | Left Superior Medial Gyrus | 5 | -33 | 41 |
| Outcome Explode - Outcome Inflate modulated | Right Supra-Marginal Gyrus | -68 | 24 | 21 |
|  | Left Superior Temporal Gyrus | 53 | 14 | 13 |
|  | Left Postcentral Gyrus | 20 | 37 | 66 |
|  | Right Middle Occipital Gyrus | -33 | 82 | 18 |
|  | Right Cerebellum Lobule VIIa | -35 | 84 | -27 |
|  | Left Middle Temporal Gyrus | 60 | 42 | 11 |
|  | Right Precentral Gyrus | -43 | 9 | 41 |
|  | Left Inferior Frontal Gyrus (p. Orbitalis) | 45 | -28 | -12 |
|  | Right Caudate Nucleus | -13 | -8 | -10 |
|  | Left Postcentral Gyrus | 40 | 17 | 38 |
|  | Left Rectal Gyrus | 3 | -56 | -15 |
|  | Left Middle Cingulate Cortex | 3 | 9 | 41 |
|  | Left Putamen | 15 | -6 | -7 |
|  | Left Putamen | 33 | 7 | 6 |
|  | Right Lingual Gyrus | -10 | 74 | 3 |
|  | Left Postcentral Gyrus | 43 | 12 | 43 |
|  | Right Cerebellum Lobule VIIIb | -20 | 42 | -50 |
|  | Right Superior Occipital Gyrus | -23 | 102 | 6 |
|  | Left Precentral Gyrus | 35 | 22 | 58 |
|  | Right Cerebellum Lobule VIIb | -35 | 62 | -52 |
|  | Left Middle Occipital Gyrus | 25 | 102 | 1 |
|  | Left Middle Frontal Gyrus | 28 | -51 | 23 |
|  | Left Middle Frontal Gyrus | 23 | -41 | 33 |
| Outcome Explode - Outcome Win unmodulated | Right Inferior Occipital Gyrus | -30 | 94 | -2 |
|  | Left Lingual Gyrus | 10 | 79 | -2 |
|  | Left Precentral Gyrus | 40 | 22 | 61 |
|  | Right Precentral Gyrus | -45 | -8 | 36 |
|  | Left Middle Occipital Gyrus | 33 | 94 | -10 |
|  | Right Cerebellum Lobule VIIIa | -28 | 54 | -55 |
|  | Right Insula Lobe | -43 | -21 | -10 |
|  | Left Insula Lobe | 33 | -21 | -10 |
|  | Left Inferior Parietal Lobule | 28 | 52 | 48 |
|  | Left Supplementary Motor Area | -3 | -23 | 46 |
| Outcome Inflate - Outcome Win unmodulated | Right Lingual Gyrus | -10 | 72 | -2 |
|  | Left Middle Cingulate Cortex | 8 | 9 | 46 |
|  | Left Middle Temporal Gyrus | 60 | 57 | 8 |
|  | Right Postcentral Gyrus | -28 | 39 | 63 |
|  | Right Lingual Gyrus | -20 | 99 | -7 |
|  | Right Inferior Frontal Gyrus (p. Triangularis) | -50 | -31 | 6 |
|  | Left Postcentral Gyrus | 38 | 17 | 41 |
|  | Right Precentral Gyrus | -38 | 17 | 41 |
|  | Right Putamen | -33 | 12 | -2 |
|  | Left Middle Occipital Gyrus | 28 | 99 | 1 |
|  | Left Middle Temporal Gyrus | 58 | -1 | -15 |
|  | Left Inferior Frontal Gyrus (p. Orbitalis) | 43 | -23 | -12 |
|  | Right Medial Temporal Pole | -55 | -16 | -25 |
|  | Left Anterior Cingulate Cortex | 5 | -36 | 6 |
|  | Left Inferior Frontal Gyrus (p. Triangularis) | 53 | -26 | 3 |
|  | Left Cerebellum Lobule VIIIb | 15 | 62 | -55 |
|  | Right Middle Frontal Gyrus | -30 | -33 | 41 |
|  | Left Putamen | 25 | 2 | -10 |
|  | Left Postcentral Gyrus | 58 | 7 | 21 |
|  | Right Amygdala | -28 | 4 | -12 |
|  | Right Thalamus | -18 | 24 | 6 |
|  | Left Putamen | 30 | -6 | 6 |
|  | Right Rolandic Operculum | -43 | 14 | 23 |

References

1. Cox RW: AFNI: software for analysis and visualization of functional magnetic resonance neuroimages. Comput Biomed Res. 1996. 29:162–73

2. Schmidt SNL, Sehrig S, Wolber A, Rockstroh B, Mier D: Nothing to lose? Neural correlates of decision, anticipation, and feedback in the balloon analog risk task. Psychophysiology. 2024. 61:e14660

3. Chen G, Adleman NE, Saad ZS, Leibenluft E, Cox RW: Applications of multivariate modeling to neuroimaging group analysis: a comprehensive alternative to univariate general linear model. Neuroimage. 2014. 99:571–88

4. Schaefer A, Kong R, Gordon EM, Laumann TO, Zuo XN, Holmes AJ, Eickhoff SB, Yeo BTT: Local-Global Parcellation of the Human Cerebral Cortex from Intrinsic Functional Connectivity MRI. Cereb Cortex. 2018. 28:3095–114

5. Fonov V, Evans AC, Botteron K, Almli CR, McKinstry RC, Collins DL: Unbiased average age-appropriate atlases for pediatric studies. NeuroImage. 2011. 54:313–27

6. Couvy-Duchesne B, Ebejer JL, Gillespie NA, Duffy DL, Hickie IB, Thompson PM, Martin NG, de Zubicaray GI, McMahon KL, Medland SE, Wright MJ: Head Motion and Inattention/Hyperactivity Share Common Genetic Influences: Implications for fMRI Studies of ADHD. PLoS One. 2016. 11:e0146271; Srichawla BS, Telles CC, Schweitzer M, Darwish B: Attention Deficit Hyperactivity Disorder and Substance Use Disorder: A Narrative Review. Cureus. 2022. 14:e24068; Thomson P, Loosley V, Friedel E, Silk TJ: Changes in MRI head motion across development: typical development and ADHD. Brain Imaging Behav. 2024. 18:1144–52

7. Chén OY, Crainiceanu C, Ogburn EL, Caffo BS, Wager TD, Lindquist MA: High-dimensional multivariate mediation with application to neuroimaging data. Biostatistics. 2018. 19:121–36

8. Friedman J, Hastie T, Tibshirani R: Regularization Paths for Generalized Linear Models via Coordinate Descent. J Stat Softw. 2010. 33:1–22

9. Friedman JH, Hastie T, Tibshirani R: Regularization Paths for Generalized Linear Models via Coordinate Descent. Journal of Statistical Software. 2010. 33:1 – 22

10. DeLong ER, DeLong DM, Clarke-Pearson DL: Comparing the areas under two or more correlated receiver operating characteristic curves: a nonparametric approach. Biometrics. 1988. 44:837–45

11. Robin X, Turck N, Hainard A, Tiberti N, Lisacek F, Sanchez J-C, Müller M: pROC: an open-source package for R and S+ to analyze and compare ROC curves. BMC Bioinformatics. 2011. 12:77

12. Kuhn M: Building Predictive Models in R Using the caret Package. Journal of Statistical Software. 2008. 28:1 – 26
